# Novel G-protein-coupled receptor-like proteins in the plant pathogenic fungus *Magnaporthe grisea*

**DOI:** 10.1186/gb-2005-6-3-r24

**Published:** 2005-03-02

**Authors:** Resham D Kulkarni, Michael R Thon, Huaqin Pan, Ralph A Dean

**Affiliations:** 1 Fungal Genomics Laboratory, Center for Integrated Fungal Research, North Carolina State University, Raleigh, NC 27695, USA; 2 Current address: Bioinformatics Program, Research Computing Division, RTI International, 3040 Cornwallis Road, Research Triangle Park, NC 27707, USA; 3 Program for Biology of Filamentous Fungi, Department of Plant Pathology and Microbiology, Texas A&M University, College Station, TX 77843, USA

## Abstract

**Background:**

The G-protein-coupled receptors (GPCRs) are one of the largest protein families in human and other animal genomes, but no more than 10 GPCRs have been characterized in fungi. Do fungi contain only this handful or are there more receptors to be discovered? We asked this question using the recently sequenced genome of the fungal plant pathogen *Magnaporthe grisea*.

**Results:**

Proteins with significant similarity to fungus-specific and other eukaryotic GPCRs were identified in *M. grisea*. These included homologs of known fungal GPCRs, the cAMP receptors from *Dictyostelium*, and a steroid receptor mPR. We also identified a novel class of receptors typified by PTH11, a cell-surface integral membrane protein required for pathogenicity. PTH11 has seven transmembrane regions and an amino-terminal extracellular cysteine-rich EGF-like domain (CFEM domain), a characteristic also seen in human GPCRs. Sixty-one PTH11-related proteins were identified in *M. grisea *that shared a common domain with homologs in *Neurospora crassa *and other fungi belonging to this subphylum of the Ascomycota (the Pezizomycotina). None was detected in other fungal groups (Basidiomycota or other Ascomycota subphyla, including yeasts) or any other eukaryote. The subclass of PTH11 containing the CFEM domain is highly represented in *M. grisea*.

**Conclusion:**

In *M. grisea *we identified homologs of known GPCRs and a novel class of GPCR-like receptors specific to filamentous ascomycetes. A member of this new class, PTH11, is required for pathogenesis, thus suggesting roles in pathogenicity for other members. The identified classes constitute the largest number of GPCR-like proteins reported in fungi to date.

## Abstract 2

An analysis of the *Magnaporthe grisea* genome and comparison with other fungi identified homologs of known G protein-coupled receptor-like proteins and a novel class of GPCR-like receptors in *M. grisea* that are specific to filamentous ascomycete fungi.

## Background

Cell-surface G-protein-coupled receptors (GPCRs) bind exogenous as well as endogenous ligands such as photons, odorants, lipids, nucleotides, hormones, pheromones, peptides and proteins. Interaction with these ligands drives diverse processes such as photoreception, taste and olfactory sensations in animals, mating in fungi and cell-cell communications in slime molds [1-3]. These receptors are characterized by seven transmembrane α-helices that upon ligand binding relay the signal by bringing about conformational changes in bound G proteins. The extracellular amino terminus in most cases interacts with the ligand and the carboxyl terminus with G proteins. The G proteins in turn activate different signaling pathways, such as those activated by adenylate cyclase and phospholipase C. These GPCRs are of immense importance as they are major targets for drug discovery [4].

A classification scheme that encompasses all GPCRs is the grouping into classes A-E [5]. A-C are the main classes present in animals: class A is the largest and comprises the rhodopsin-like receptors, class B comprises the secretin-like receptors and class C the metabotropic glutamate/pheromone receptors. Class D is unique to fungi and comprises fungal pheromone receptors. Class E contains cAMP receptors, such as the cAMP receptors of *Dictyostelium*. Other classes include frizzled/smoothened, adhesion receptors and the insect-specific chemosensory receptors [6,7]. Sequence conservation between GPCR classes is limited, however, with each receptor class exhibiting specific identifiable characteristics [6,8]. The secretin and the adhesion receptors are characterized by conserved cysteine residues or by known cysteine-rich domains resembling the epidermal growth factor (EGF) domain at their amino termini.

GPCRs form the largest family of receptors in animals, with more than 600 members in the human genome [9,10]. Only a handful of GPCRs have been identified in fungal genomes, however. In *Saccharomyces cerevisiae *and *Schizosaccharomyces pombe *only three and four receptors, respectively, are well characterized [1,11-16]. In the *Neurospora crassa *genome a total of 10 receptors is predicted [17]. A recent report for *Aspergillus nidulans *identified GPCRs similar to the yeast pheromone receptors, the glucose-sensing receptor GPR1, the nitrogen-starvation sensing STM1, and the *Dictyostelium discoideum *cAMP receptors [18]. Given the prevalence and significance of GPCRs in higher eukaryotes, their relative paucity in the kingdom Fungi warranted further investigation. To see if we could find additional families, we searched the predicted proteome of the rice blast fungus *Magnaporthe grisea*.

The fungal plant pathogen *M. grisea *is a powerful model system to study the pathogenicity determinants required for plant cell-surface recognition and production of an appressorium, a specialized structure required to penetrate the plant surface [19,20]. The fungus causes rice blast disease, the most destructive disease of rice worldwide. *M. grisea *is amenable to molecular genetic manipulation and the subject of large-scale genome-wide functional studies following the recent completion of a draft genome sequence [21]. Infection begins when a conidium, attached to the plant surface, sends forth a germ tube that differentiates to form a highly melanized appressorium. Turgor pressure inside the appressorium results in a penetration hypha breaching the cell wall and invasion of the plant tissues. This developmental program, which is accompanied by a number of biochemical and developmental changes, is a result of perception by the fungus of appropriate environmental and plant cell-surface signals and induction of a cascade of signaling pathways.

Cell-surface receptors that perceive signals at critical times in the life cycle of *M. grisea *and other pathogenic fungi are strongly implicated as pathogenicity determinants. Signaling plays a key role in appressorium formation and infection in *M. grisea*. The cAMP-dependent and pheromone response, as well as other mitogen-activated protein kinase (MAPK)-, phospholipase- and calmodulin-dependent pathways, are essential for pathogenicity and are likely to involve perception of signals through GPCRs [22-24]. The three identified G-protein alpha subunits, required for different aspects of development and pathogenicity, possibly transduce perceived signals to the above-mentioned pathways [25]. The *M. grisea *G proteins probably receive signals from receptors such as PTH11, an integral membrane protein required for pathogenicity [26]. As animal GPCRs are important targets for drug discovery, identifying fungal receptors would be equally important for understanding and controlling *M. grisea *and other fungal pathogens.

Identification of new GPCR classes is difficult because of low sequence similarity; even within related classes, sequence conservation is limited to the membrane-spanning regions [8]. There are also large variations in the type and number of receptors in classes that show no sequence or structural similarities to each other. We therefore carried out an exhaustive analysis to mine the proteome of the sequenced genome of the rice blast fungus *M. grisea *for GPCR-like proteins. Homologs of known fungal GPCRs were found in the *M. grisea *proteome, including the pheromone receptors STE2 and STE3 and the glucose-sensing receptor GPR1. In total, 76 GPCR-like proteins were identified in the present study of which 61 represent a large novel class related to PTH11, a receptor implicated in fungal development and pathogenicity and proposed to act upstream of the cAMP-dependent pathway. Many of these novel receptors will have roles in known pathways or may define new pathways involved in fungal development.

## Results

### Identification of novel classes of GPCR-like proteins in *M. grisea*

We searched the *M. grisea *proteome for GPCR-like proteins on the basis of their similarity to known receptors. GPCR sequences including all present in the GPCR database (GPCRDB [5]) were used as a query in a BLASTP search against the *M. grisea *predicted protein set [21]. The proteins retrieved in this search were used to BLAST the *M. grisea *proteins again to find all related sequences (Table 1). A total of 14 GPCR-like proteins were found. These included homologs of characterized fungal GPCRs (GPR1, STM1, and the STE2- and STE3-like pheromone receptors). Other proteins identified were similar to the cAMP receptors and to mPR, a steroid receptor. No homologs of the animal rhodopsin-, secretin- and metabotropic-like receptor classes, which form the majority of the proteins in GPCRDB, could be found. All proteins listed in the table were checked to make sure they had seven transmembrane regions (Additional data file 1). The *M. grisea *proteins were searched with InterProScan [27] and 16 proteins associated with InterPro entries containing the terms 'GPCR' or 'G protein-coupled receptors' were identified. Four were already identified in the above BLAST searches. Of the remaining 12, only one (MG00532.4) had seven transmembrane regions and was added to Table 1. This protein had weak similarity to rat growth hormone-releasing factor receptor and other GPCRs. A PfamA HMM search revealed that some of the proteins identified above had characteristic GPCR domains (Table 1, and see [28]).

The receptor PTH11 in *M. grisea *is required for development of the appressorium [26]. It is an integral membrane protein and has been localized to the cell membrane. It is proposed to act upstream of the cAMP pathway, which is required for pathogenicity. The PTH11 amino-terminal domain contains an EGF-like cysteine rich CFEM domain, predicted to be extracellular, followed by seven transmembrane regions [29]. Based on the transmembrane topology, with the amino-terminal outside and the carboxy-terminal inside, PTH11 is a novel GPCR-like protein. PTH11 has been reported to have nine transmembrane regions; however, the two putative transmembrane regions at the amino-terminal end are the predicted signal sequence and the hydrophobic region within the extracellular CFEM domain, respectively, and are therefore not membrane spanning [26,29]. The CFEM domain is an EGF-like domain, characteristically present in the extracellular regions of membrane proteins; thus PTH11 is characterized as having an extracellular amino-terminal CFEM domain, followed by seven transmembrane regions. A BLASTP search using PTH11 as query against known *M. grisea *proteins retrieved a number of proteins with seven transmembrane regions (E-value cutoff of 1e-09). A BLASTP search using these PTH11-related proteins against *M. grisea *predicted proteins returned additional members within this class (total 61, Table 1). Only a subset of the retrieved proteins contained the CFEM domain, as indicated in Table 1 (12 CFEM-containing proteins).

In total we identified 76 receptors, including members of known classes as well as novel classes. Sixty-one represented a novel class that included PTH11. All other receptors identified were assigned to different classes on the basis of their similarity to known receptors using BLASTP against the GenBank (nonredundant) and Swiss-Prot databases, and their conserved domain characteristics. We found three members of the mPR class and one (MG0532.4) with weak similarity to animal GPCRs. No members of these classes have been reported previously in fungi. Within each class, members were assigned to paralogous families (Table 1). Many of the genes in Table 1 are expressed, as suggested by representation in expressed sequence tags or microarray experiments. A BLAST search against the GenBank EST databases revealed that some of the predicted open reading frames (ORFs) had matches in the *M. grisea *ESTs (Table 1, and see [30]). Results from microarray experiments on gene expression during conidia germination and appressorium formation also showed that many of these ORFs are expressed (T.K. Mitchell and R.A.D, unpublished work).

### Shared and unique GPCR-like protein classes in *M. grisea*

*M. grisea *GPCRs were compared with published fungal genome sequence databases to identify proteins belonging to the same GPCR classes. A BLASTP search against the genome of the closely related filamentous fungus *N. crassa *[17], using all the *M. grisea *GPCR-like proteins as query, revealed the presence of similar proteins in *N. crassa*, including PTH11 homologs (Table 2 and Additional data file 2). No PTH11 homologs were found in *S. cerevisiae *and *S. pombe*. Further analysis revealed putative homologs of the mPR-1 class in both yeasts, in which they had not previously been identified. In addition, we found no evidence for cAMP receptor-like GPCRs in either yeast, unlike both *M. grisea *and *N. crassa*. The cAMP, STM1, and mPR receptors are shared between fungi and other eukaryotic species. However, the fungal pheromone receptors (class D) and GPR1-like receptors appear to be fungus-specific.

Members of the large class of PTH11-related receptors were restricted to a fungal subphylum. BLASTP of all the PTH11 class members, and PSI-BLAST using conserved regions, against the GenBank (nonredundant) and Swiss-Prot databases and publicly available fungal genomes retrieved matches in members of the subphylum Pezizomycotina within the Ascomycota, including *Podospora anserina*, *Blumeria graminis*, *Fusarium graminearum *and *Aspergillus *species. Other fungi belonging to the Ascomycota but not to this subphylum, such as *S. cerevisiae*, *S. pombe*, *Candida albicans *and *Pneumocystis carinii *lacked PTH11-related sequences. Also, no PTH11-related sequences were found in the genomes of the Basidiomycetes *Cryptococcus neoformans*, *Ustilago maydis *and *Phanerochaete chrysosporium*. No matches were found in plant, animal or prokaryotic genomes.

### Phylogenetic analysis of PTH11-related GPCR-like proteins in *M. grisea *and *N. crassa*

**Figure 1:**
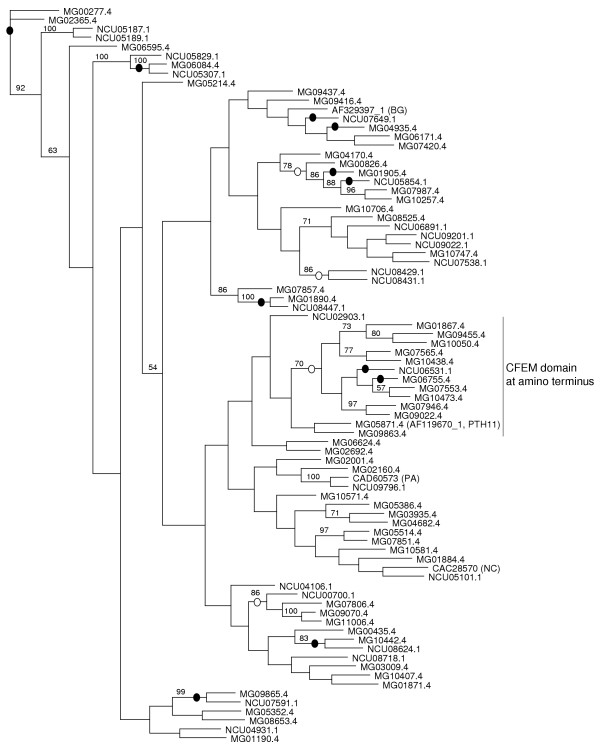
Gene phylogeny based on the conserved membrane-spanning PTH11-domain. The tree shown was constructed using parsimony methods. Numbers on branches represent bootstrap values based on 100 random dataset simulations. Open ovals indicate putative paralogs and filled ovals the *M. grisea*-*N. crassa *orthologs. For sequences other than the ones predicted from *M. grisea *and *N. crassa *genome sequences the GenBank accession numbers are indicated. The abbreviations for species names are indicated in parentheses after the accession numbers as follows: BG, *Blumeria graminis*; PA, *Podospora anserina*; NC, *N. crassa*. The product of the gene *PTH11 *was referred to as Pth11p in the original report. Subsequently it has been referred to as PTH11. We refer to this gene product as PTH11 in this paper and would like to propose revision of its name from Pth11p to PTH11.

**Figure 2:**
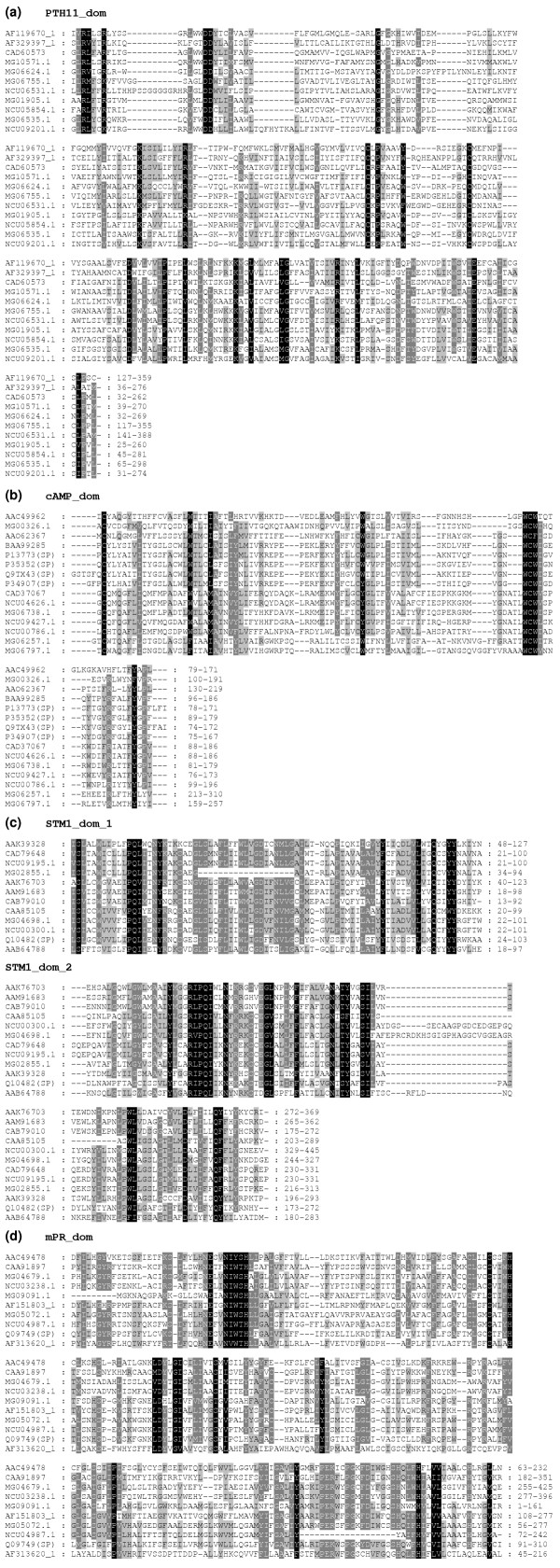
Alignment of GPCR-like proteins. Domains conserved in **(a) **PTH11-, **(b) **cAMP-, **(c) **STM1- and **(d) **mPR-related classes are shown. Representative sequences from each class were aligned using T_Coffee [39]. The alignment was analyzed using GenDoc. We used the default setting using the conservative shading mode with similarity groups enabled. Black and the dark and light gray represent 80% or greater conserved, 60% or greater conserved, and less than 60% conserved, respectively. Conservative substitutions were counted as a single residue type. The GenBank or Swiss-Prot (SP) accession numbers or the accession numbers of the predicted proteins in the *M. grisea *or *N. crassa *genome databases are indicted on the left [21, 42]. The boundaries of each sequence used in the alignment are indicated on the right.

PTH11-related receptors from *M. grisea *and *N. crassa *were classified into paralogous families (Additional data file 2). We also identified any that were orthologs between these two species. PTH11-related receptors in *M. grisea *and *N. crassa *and other sequences from *P. anserina *and *B. graminis *were aligned to determine any relationships. The region containing the conserved PTH11-domain was used to build a phylogenetic tree (Figures 1, 2a). Our analysis indicated that PTH11-related proteins form a large and divergent protein family that evolved before the divergence of *M. grisea *and *N. crassa*. *M. grisea *and *N. crassa *orthologs occurred in the same clades (Figure 1). Many different clades on the tree may represent paralogous sequences. The tree supports the putative orthologs and paralogs we identified (see Additional data file 2). Even though only the PTH11 domain was used to build the tree (the amino-terminal CFEM domain seen in a few proteins was not included), the 13 CFEM domain-containing proteins occurred together in one clade, indicating that the sequences are closely related. The phylogeny also revealed that within certain clades there was a marked expansion of the PTH11-related proteins in *M. grisea *compared to *N. crassa*. This is particularly notable for the CFEM domain-containing proteins. There were six *M. grisea *members containing the CFEM domain in a paralogous family (Table 1 and Figure 1; a total of 12 related CFEM and seven-span proteins), but only one from *N. crassa*. We found 38 PTH11-related proteins in *A. nidulans *with an E-value less than 1e-09. Further characterization of these proteins will be required to define the number of seven-span PTH11-related proteins in this genome. Preliminary analysis shows that only two seven-span proteins contain the CFEM domain in *A. nidulans*. These observations could represent either expansion since speciation of the CFEM-containing PTH11 relatives in *M. grisea*, or loss of these proteins in the other fungal species.

### New domain signatures as defined by conserved regions in homologous classes of identified receptors

Members of each class of *M. grisea *GPCR-like proteins described above, for example, cAMP-, STM1-like, PTH11-related receptors, have domains that are conserved within each class. Sequence alignments from the BLASTP searches revealed specific regions containing shared residues for each of these classes of receptors. Figure 2 shows an alignment for some of the sequences that belong to classes other than the better-studied pheromone and glucose-sensing receptors. In all the PTH11-related members the region towards the amino terminus was conserved (Figure 2a, PTH11_dom). The extreme amino-terminal and the carboxy-terminal sequences flanking this region were divergent. Conserved residues occurred within the seven-span regions for all of these proteins. This is consistent with other observations that sequence conservation is typically limited to the transmembrane regions in GPCRs. The *M. grisea *protein MG06738.4, which has similarity to the cAMP receptors, shared conserved amino-acid residues between positions 81-179 with MG06797.4, MG00326.4, MG06257.4, related *N. crassa *proteins and other cAMP receptors (cAMP_dom, Figure 2b). Other proteins - MG00258.4 and MG10544.4 - with weak similarity to cAMP receptors also shared residues within this domain (data not shown). MG04698.4 shared two domains between amino-acid residues 22-101 and 244-327 with STM1, MG02855.4 and related proteins from different eukaryotic species (stm1_dom, Figure 2c). MG05072.4 shared residues within the region of 56-277 with MG09091.4 (residues 18-228), MG04679.4 (residues 260-497) and other proteins that were retrieved in the BLAST search, including mPR receptors (mPR_dom, Figure 2d).

The proteins containing the PFAM GPCR domains are indicated in Table 1. It is worth noting that the low scores for the PFAM domains that we observed may be due to the need to update these domain alignments by adding many new proteins, including those we discovered. For example, MG06452.4 contains a putative STE3 domain; the alignment score (E-value) is low, however. With the new fungal genomes being sequenced, more STE3 homolog sequences are available and inclusion of these in the seed alignment defining the STE3 domain will make the domain more representative for fungal STE3 domains.

**Figure 3:**
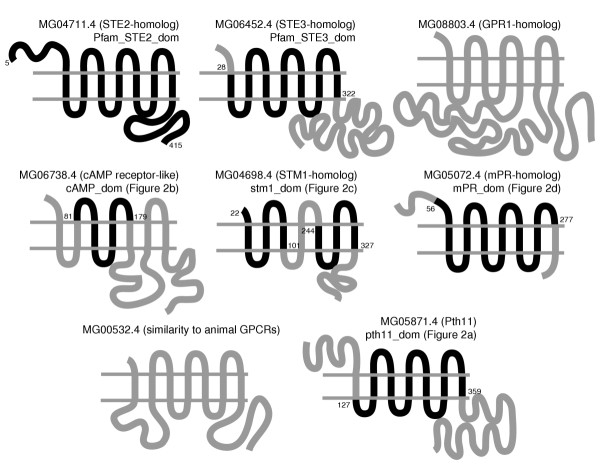
Membrane topology of *M. grisea *GPCR-like proteins. The figure shows representative examples from different classes with domains that are conserved with respect to other receptors of the same class. Known Pfam domains or domains conserved between the *M. grisea *protein and other members of the class, as shown in Figure 2, are shaded in black. The amino-acid residue numbers that mark the boundaries of these domains are given. The location of the domains on the membrane topology shown for the *M. grisea *protein is the same for other proteins that share these domains. For GPR1-related proteins, sequence similarity was limited to the membrane-spanning regions and MG00532.4 had sequence similarity with other animal GPCRs between the third and the fifth membrane-spanning regions (not shown in figure).

Each class of receptors contained specific conserved regions within the membrane-spanning topology. A representative example of each class, showing the location of the conserved region within the membrane topology is illustrated in Figure 3. For fungus-specific receptors, the conserved domain spanned almost the entire length of the seven transmembrane regions. When other eukaryotic receptors were included in the class, however, only shorter conserved domains were discerned. These conserved residues may reflect functional constraints and may be valuable for studying the structure-function relationships of these proteins.

## Discussion

### Distinct classes of GPCR-like proteins identified in *M. grisea*

Fungi respond to a variety of signals from the environment that regulate cellular metabolism and development as well as host-pathogen interactions. Cell-surface receptors perceive these signals and relay them to intracellular signaling pathways. We searched the proteome of *M. grisea *for GPCR-like proteins and identified a total of 76 sequences (Table 1). This is the largest number of GPCR candidates identified for any fungal species. The identified proteins in *M. grisea *include homologs of known fungal receptors and a few other eukaryotic receptors. Putative orthologs of fungal STE2- and STE3-like pheromone receptors required for the mating responses in yeast were identified. A homolog of GPR1, which is involved in pseudohyphal differentiation in *S. cerevisiae*, and two proteins that share similarities with STM1 from *S. pombe *were also found [11,13,16]. Six proteins shared similarities with cAMP receptors from *Dictyostelium*. In *Dictyostelium *the cAMP receptors are involved in establishing polarity during chemotaxis [3]. All the above *M. grisea *proteins can be annotated as putative GPCRs on the basis of homology to known receptors. It is likely that they respond to similar ligands, such as pheromones, nutrients and cAMP (Table 1). Response to fungal mating pheromones and the existence of pheromone receptors in *M. grisea *was first suggested by the observation that *M. grisea *responded to *S. cerevisiae *pheromones in a mating-type-specific manner [22]. Intracellular cAMP, produced by adenylate cyclase, is a critical factor regulating appressorium development in *M. grisea*. Lee and Dean have found that the fungus will respond to exogenously added cAMP by development of appressoria, although the concentrations required are high [31]. They noted that the cell wall and cell membrane should be relatively impermeable to cAMP, and thus any responses to extracellular cAMP will be due to cAMP receptors. Further research will be required to learn about the mechanism of perception of exogenous cAMP and other ligands and their targets within the cell.

PTH11-related proteins share a number of characteristics diagnostic of GPCRs and define a new class of GPCR-like proteins. The predicted membrane topology suggests a seven-span protein with an amino terminus outside the cell, that could respond to extracellular signals, and a cytoplasmic carboxy-terminal domain that could interact with G proteins. All the PTH11-related proteins shared conserved residues within the membrane spans, as observed in other GPCRs classes [8]. A subclass of the PTH11 receptors showed another characteristic that is seen in a few classes of human GPCRs: they have an amino-terminal cysteine-rich EGF-like CFEM domain. The animal secretin receptors are characterized by six conserved cysteines at the amino terminus, with cysteine bridges implicated in ligand binding. Some of the adhesion receptors have cysteine rich-EGF-like domains at their amino termini [6,8]. CFEM-domain-containing proteins, which are smaller in size and lack the seven transmembrane regions, may interact with the CFEM-containing GPCR-like proteins (Additional data file 3 and [29]). The CFEM-containing proteins have a signal peptide and/or a glycosylphosphatidylinositol (GPI) anchor. Thus they are either secreted from the cell or are anchored to the cell membrane. They may be similar to the odorant-binding proteins, which also have cysteine-rich domains and have been proposed to interact with odorant-GPCRs [32].

### Unique classes of fungal G-protein-coupled receptors with ancient origins

Having diverged approximately 1,460 million years ago (Mya) [33], it is clear that fungi have classes of GPCRs that are distinct from those of animals. The class D fungal pheromone receptors define a fungus-specific class of receptors. We found the GPR1-like receptors to be also fungal specific. Classes of receptors specific to a group of species also occur in animals. For example, some of GPCRs in *Anopheles gambiae *constitute an insect-specific class of chemosensory receptors [7]. Insects are estimated to have diverged from other animals nearly 1,000 Mya. Thus, we would expect to find novel fungal GPCRs with no similarities to ones present in other eukaryotic kingdoms. The largest class of *M. grisea *GPCR-like proteins we identified is the novel PTH11-related class. It is interesting that we only found homologs of PTH11 in fungi belonging to subphylum Pezizomycotina within the Ascomycota (this subphylum has an estimated divergence date of 1,140 Mya). None was found in fungi belonging to other subphyla in Ascomycota or Basidiomycota, estimated to have diverged from each other 1,210 Mya. This indicates that these proteins are extremely ancient in origin, having possibly evolved to serve specialized functions in a specific subgroup of fungi. They are either unique to this fungal group or have evolved sufficiently to be unrecognizable.

### Relationships between the PTH11-related proteins

The PTH11-related proteins form a large and divergent protein family, as suggested by the similarity between the proteins and the phylogenetic tree (Table 1, Figure 1). This gene family may have evolved before the divergence of *M. grisea *and *N. crassa*. There are a few orthologs between these species; however, it is apparent that this family has undergone considerable expansion in *M. grisea *compared to *N. crassa*, with the largest subclass in *M. grisea *being the CFEM-containing proteins. Many of the *PTH11*-related genes are located in close proximity to each other on the genome (data not shown), whereas none of the other GPCR-like proteins, except a pair of cAMP-receptor-related proteins, occurs in close proximity. A paralogous pair, MG07553.4 and MG07565.4, occurs close together on linkage group III, indicating that these genes may have arisen as a result of duplication. We blasted these sequences against each other and observed that they show 30% identity with an E-value of 7e-54. This suggests that even if these genes are a result of duplication, they have diverged sufficiently and are not incorrect duplicate predictions of the same gene due to sequencing or assembly errors. Both these genes contain the CFEM domain and also occur in the same clade on the phylogenetic tree (Figure 1). Another pair of CFEM-containing proteins is located in close proximity (LGI, group 1). The above examples of relative expansions within the PTH11-related proteins, as compared to *N. crassa*, are an indication that gene duplication may still be occurring in *M. grisea*. In *N. crassa *it is believed that because of the phenomenon of repeat induced point mutations (RIP), gene duplications are not maintained [17]. There is evidence of RIP in *M. grisea*, but the present study provides an example that has escaped the RIP process [34]. Other possibilities are that these genes duplicated before the evolution of RIP or have escaped RIP because *M. grisea *rarely undergoes meiosis in the wild.

### Regulation of the activity of GPCR-like proteins by differential expression and interaction with different signal transducers

Differential expression and interaction with different signal transducers could be a way to regulate specific signaling pathways. Results from genome-wide microarray experiments suggest different patterns of expression for the GPCR-like receptors during growth and development (T.K. Mitchell and R.A.D, unpublished work). Representation of some of the GPCR-like receptors in the fungal ESTs and microarray experiments suggests that most of these genes are expressed (Table 1). In addition to differential regulation of the GPCR-like proteins, their interaction with different G proteins could channel various signals to different pathways. As well as the well studied interactions with G proteins, it has been proposed that the seven-span receptors may also interact with other signal transducers and receptor-interacting proteins to transmit the signal to different cellular pathways.

## Conclusion

The number of classes of GPCR-like proteins identified in the present study is the largest reported in fungi. Further research on these receptors will help delineate potentially novel signaling pathways with which they interact. The new class of PTH11-related receptors, specific to an Ascomycota subphylum and relatively numerous in *M. grisea*, is particularly interesting. PTH11 is an integral membrane protein localized to the cell membrane and is required for pathogenicity [26]. It is proposed to act upstream of the cAMP pathway as a receptor that channels signals to this pathway. PTH11 does not have an ortholog in *N. crassa*. Also, as discussed earlier, only one CFEM-containing seven-span protein is present in *N. crassa *compared to 12, including PTH11, in *M. grisea*. It remains to be determined whether other members of this expanded class of PTH11-related proteins are involved in different aspects of pathogenicity. The subphylum Pezizomycotina includes the majority of known ascomycete species, and includes pathogens and mutualists. Because PTH11-related GPCR-like proteins are present in non-pathogens, many members of this class are likely to be involved in functions not related to pathogenesis. All the seven-span receptors and their characteristic domain signatures we discovered (Figures 2, 3) will be valuable in the identification and comparative studies of new receptors in the many fungal genomes being sequenced.

## Materials and methods

### Identification of GPCR-like proteins in *Magnaporthe grisea*

Known GPCR sequences, including ones present in the GPCRDB [5], were BLASTed against the predicted *M. grisea *proteome to identify homologs in *M. grisea *[21]. The database containing 7,900 GPCR sequences (updated 28 May 2003) was used as a query in a BLASTP search against the *M. grisea *predicted proteins with an E-value limit of 1e-09. Results from an InterPro scan of the *M. grisea *proteins were searched for domains containing the following terms: 'GPCR' and 'G-protein-coupled receptors' [27]. *M. grisea *PTH11, a GPCR-like protein (see Results), was also used in a BLASTP search against the *M. grisea *proteome. BLAST and PfamA searches and related sequence analysis were done using Genomax (Informax (now Invitrogen)).

### Characterization of the GPCR-like proteins and identification of additional members in *M. grisea *and other fungi

GPCR-like sequences were evaluated for seven transmembrane regions by TMPRED, Phobius and TMHMM [35-37]. Default settings were used. In nearly all cases at least two of the algorithms predicted the seven-span helix topology (Additional data file 1). A BLASTP search using the seven-span polypeptide sequences as query against the *M. grisea *protein set was also done to identify any other similar members. The set of identified seven-span proteins was then subject to BLASTP analysis against GenBank and Swiss-Prot to confirm sequence similarity to GPCRs. This exercise also allowed identification of other members that were similar to these sequences. The *M. grisea *seven-span proteins identified as above were used as a query in a BLAST search against the *N. crassa *predicted proteins [17] to identify homologs. The *M. grisea *and *N. crassa *proteins were placed into clusters using the blastclust program [38]. All *M. grisea *and *N. crassa *proteins that had at least 30% identity and 80% overlap over the length of the proteins were clustered together. Members of the same species within a cluster were considered paralogs. Orthologs were defined as proteins that had bidirectional best BLAST hits. A TBLASTN search using the seven-span containing sequences as query against the GenBank EST database was performed to identify any identical matches in the *M. grisea *ESTs (or other closely related fungal sequences). The GPCR-like sequences identified in *M. grisea *were used as query in BLASTP searches (cutoff < 1e-09) against the *S. cerevisiae *and *S. pombe *genomes and other completely sequenced fungal genomes to identify putative homologs in these species.

### Alignments and phylogenetic relationships between the predicted GPCR sequences

The alignment of sequences within related classes in Figure 2 was done using T_Coffee and minor editing as per results from the BLAST alignments was done using GenDoc [39]. For phylogenetic analysis, the conserved PTH11-domain that spans the membrane-spanning regions was used. Sequences were aligned using ClustalW version 1.81 [40]. The phylogenetic tree was constructed using PAUP by both neighbor-joining and parsimony methods followed by bootstrap analysis (100 bootstrap replications). A tree was also constructed using the neighbor-joining method implemented in the software package MEGA 2.1 [41]. All methods showed similar relationships between the proteins.

## Additional data files

The following additional data is available with the online version of this paper: additional data file 1 is a table listing *M. grisea*-GPCR-like protein accession numbers and seven-span predictions; additional data file 2 is a table listing *M. grisea*-GPCR-like protein classes and N. *crassa *homologs; additional data file 3 is a table listing *M. grisea *CFEM-containing proteins that may be membrane associated or secreted.

## Supplementary Material

Additional File 1 *M. grisea*-GPCR-like protein accession numbers and seven-span prediction. Click here for file

Additional File 2 *M. grisea*-GPCR-like protein classes and N. *crassa *homologs. Click here for file

Additional File 3 *M. grisea *CFEM-containing proteins that may be membrane associated or secreted. Click here for file

## Figures and Tables

**Table 1:** Predicted G-protein-coupled receptor-like proteins in *M. grisea*

Known receptors used as query in BLAST against *M. grisea *proteins or another search method	*M. grisea *proteins retrieved by known receptor (BLASTP)	E-value	Other proteins homologous to *M. grisea *proteins retrieved by known receptor	PfamA GPCR domains (E-value)/conserved domain identified in the present study
Pheromone receptor (CAC86431; STE2-like)	MG04711.4*	3e-65		Pfam STE2 (2.1e-04)
				
Pheromone receptor STE3 (STE3_YEAST)	MG06452.4^†^	2e-14		Pfam STE3 (1.1e-09)
				
cAMP receptor TASA (Q9NDL2)	MG06738.4* ^,†^	5e-11		Pfam7tm_2 (1.3e-04)/cAMP_dom
			MG06797.4	cAMP_dom
			MG06257.4*	cAMP_dom
				
			MG00326.4	Pfam 7tm_2 (7.9e-05)/cAMP_dom
				
			MG00258.4	cAMP_dom
				
			MG10544.4	cAMP_dom
				
GPCR GPR1 (GPR1_YEAST)	MG08803.4	4e-18		
				
GPCR STM1 (STM1_SCHPO)	MG04698.4*	5e-19		STM1_dom
				
	MG02855.4* ^,†^	1e-17		STM1_dom
				
GPCR mPR (NP_848509)	MG05072.4*	6e-17		mPR_dom
				
			MG09091.4*	mPR_dom
			MG04679.4* ^,†^	mPR_dom
				
PTH11 receptor (AF119670_1)	MG05871.4 (PTH11) * ^,†,‡^	0		PTH11_dom
				
	MG10473.4^‡^	3e-34		PTH11_dom
	MG06755.4^‡^	1e-33		PTH11_dom
	MG07553.4^‡^	2e-32		PTH11_dom
	MG09022.4* ^,‡^	2e-27		PTH11_dom
	MG07565. * ^,†,‡^4	6e-23		PTH11_dom
	MG07946.4^†,‡^	3e-21		PTH11_dom
				
	MG11006.4	2e-32		PTH11_dom
	MG09070.4*	2e-29		PTH11_dom
	MG07806.4	2e-21		PTH11_dom
	MG03584.4^†^	1e-22		PTH11_dom
				
	MG05214.4*	4e-31		PTH11_dom
	MG09863.4* ^,‡^	1e-28		PTH11_dom
				
	MG10407.4*	3e-26		PTH11_dom
				
	MG10571.4* ^,†^	4e-25		PTH11_dom
				
	MG01867.4^‡^	1e-23		PTH11_dom
				
	MG09455.4^†,‡^	2e-23		PTH11_dom
	MG10050.4^‡^	1e-14		PTH11_dom
	MG09667.4	1E-22		PTH11_dom
				
	MG05352.4*	2e-22		PTH11_dom
				
	MG07420.4	1e-21		PTH11_dom
				
	MG10442.4	4e-20		PTH11_dom
				
	MG02160.4^†^	6e-19		PTH11_dom
				
	MG02001.4* ^,†^	1e-18		PTH11_dom
				
	MG10257.4	2e-18		PTH11_dom
	MG01905.4	2e-17		PTH11_dom
	MG07987.4	1e-16		PTH11_dom
				
	MG10438.4* ^,‡^	6e-18		PTH11_dom
				
	MG06171.4*	1e-17		PTH11_dom
				
	MG07851.4	1e-17		PTH11_dom
				
	MG04935.4*	1e-17		PTH11_dom
				
	MG05386.4	3e-17		PTH11_dom
				
	MG09865.4* ^,†^	3e-16		PTH11_dom
				
	MG09061.4	4e-16		PTH11_dom
				
	MG05514.4* ^,†^	1e-16		PTH11_dom
				
	MG06535.4*	3e-14		PTH11_dom
				
	MG01190.4	7e-14		PTH11_dom
				
	MG10581.4*	7e-14		PTH11_dom
				
	MG03009.4*	2e-13		PTH11_dom
				
	MG10747.4	8e-13		PTH11_dom
				
	MG03935.4	2e-12		PTH11_dom
			MG04682.4*	PTH11_dom
				
	MG09416.4	1e-10		PTH11_dom
				
	MG02692.4*	2e-10		PTH11_dom
				
			MG07857.4	PTH11_dom
				
			MG00826.4	PTH11_dom
				
			MG06624.4* ^,†^	PTH11_dom
				
			MG00435.4*	PTH11_dom
				
			MG08653.4*	PTH11_dom
				
			MG10706.4* ^,†^	PTH11_dom
				
			MG04170.4*	PTH11_dom
				
			MG08525.4*	PTH11_dom
				
			MG00277.4*,†	PTH11_dom
			MG02365.4*	PTH11_dom
				
			MG06595.4	PTH11_dom
				
			MG06084.4*	PTH11_dom
				
			MG09437.4*	PTH11_dom
				
			MG01890.4	PTH11_dom
				
			MG01871.4	PTH11_dom
				
			MG03794.4	PTH11_dom
				
			MG01884.4*	PTH11_dom
				
InterProScan MG00532.1	MG00532.4 (weak similarity to animal GPCRs) *			Pfam 7tm_2 (1.4e-02)

Classes of GPCR-like protein in *M. grisea *were subdivided on the basis of BLASTP analysis and shared domains, as described in Materials and methods. They were clustered into paralogous families if the proteins showed 30% identity and 80% overlap over the complete length of the protein. Paralogous families are separated by a blank line. The GPCR-like proteins in *M. grisea *could be classified into nine subclasses containing more than one member and 48 containing a single member. Six subclasses contained two members, two contained three and one contained six. *M. grisea proteins represented by genes expressed in microarray experiments. ^†^*M. grisea *proteins that are represented in *M. grisea *ESTs. ^‡^Proteins containing the cysteine-rich CFEM domain.

**Table 2:** Classes of GPCR-like proteins in fungi

Class of receptors	*M. grisea*	*N. crassa*	*S. cerevisiae*	*S. pombe*
GPCR homologs of known classes				
Fungal pheromone STE2-like (class D)	1	1	1	1
Fungal pheromone STE3-like (class D)	1	1	1	1
cAMP receptor-like (class E)	6	3	-	-
				
Other GPCR homologs				
*S. cerevisiae *GPR1-like	1	1	1	1
*S. pombe *STM1-like	2	2	3*	1
*H. sapiens *mPR-like	3	2	3*	2*
*M. grisea *MG00532.4-like (weak similarities to animal GPCRs)	1	1	-	-
				
Other GPCR-like proteins				
*M. grisea *PTH11-related	61	25	-	-

*Have not been characterized as GPCR in the yeast species but do have seven transmembrane spans.
