# Supplementary material for: Novel G-protein-coupled receptor-like proteins in the plant pathogenic fungus Magnaporthe grisea
Source: Genome Biol. 2005 Mar 2;6(3):R24. doi: 10.1186/gb-2005-6-3-r24 (PMC1088943; doi:10.1186/gb-2005-6-3-r24)
Supplement: Additional File 1 — M. grisea-GPCR-like protein accession numbers and seven-span prediction. [file gb-2005-6-3-r24-S1.doc]

# Supplemental information

# Table S1. *M. grisea*-GPCR-like 7tms protein accession numbers^a^.

| *M*. *grisea* protein | GenBank Accession numbers | TMPred prediction | Phobius prediction | TMHMM prediction |
| --- | --- | --- | --- | --- |
|  |  |  |  |  |
| MG00258.4 | EAA48600 | 7tms | 7tms |  |
| MG00277.4 | EAA48619 | 7tms | 7tms |  |
| MG00326.4 | EAA48668 | 7tms |  | 7tms |
| MG00435.4 | EAA48777 | 7tms | 7tms |  |
| MG00532.4 | EAA48874 | 7tms | 7tms | 7tms |
| MG00826.4 | EAA49168 | 7tms |  |  |
| MG01190.4 | EAA55539 | 7tms | 7tms | 7tms |
| ^b^MG01867.4 | EAA56216 | 7tms | 7tms |  |
| MG01871.4 | EAA56220 | 7tms | 7tms | 7tms |
| MG01884.4 | EAA56232 | 7tms | 7tms |  |
| MG01890.4 | EAA56238 | 7tms | 7tms | 7tms |
| MG01905.4 | EAA53920 | 7tms | 7tms | 7tms |
| MG02001.4 | EAA54016 | 7tms | 7tms |  |
| MG02160.4 | EAA54175 | 7tms |  |  |
| MG02365.4 | EAA54380 | 7tms |  |  |
| MG02692.4 | EAA47449 | 7tms | 7tms | 7tms |
| MG02855.4 | EAA47612 | 7tms |  |  |
| MG03009.4 | EAA47766 | 7tms | 7tms | 7tms |
| MG03584.4 | EAA51989 |  | 7tms |  |
| MG03794.4 | EAA50035 |  | 7tms | 7tms |
| MG03935.4 | EAA50176 | 7tms | 7tms | 7tms |
| MG04170.4 | EAA50411 | 7tms | 7tms | 7tms |
| MG04679.4 | EAA50920 | 7tms | 7tms | 7tms |
| MG04682.4 | EAA50923 | 7tms | 7tms | 7tms |
| MG04698.4 | EAA50939 | 7tms | 7tms | 7tms |
| MG04711.4 | EAA50952 | 7tms | 7tms | 7tms |
| MG04935.4 | EAA52243 | 7tms |  |  |
| MG05072.4 | EAA52380 | 7tms | 7tms | 7tms |
| MG05214.4 | EAA54723 | 7tms | 7tms |  |
| MG05352.4 | EAA54560 | 7tms | 7tms | 7tms |
| MG05386.4 | EAA54594 | 7tms | 7tms | 7tms |
| ^b^MG05514.4 | EAA54723 | 7tms |  |  |
| MG05871.4 | EAA52743 | 7tms |  | 7tms |
| MG06084.4 | EAA52956 | 7tms | 7tms | 7tms |
| MG06171.4 | EAA53043 | 7tms | 7tms | 7tms |
| MG06257.4 | EAA56286 | 7tms | 7tms | 7tms |
| MG06452.4 | EAA56481 | 7tms | 7tms | 7tms |
| MG06535.4 | EAA56564 | 7tms | 7tms | 7tms |
| MG06595.4 | EAA56624 | 7tms |  |  |
| MG06624.4 | EAA56653 | 7tms | 7tms | 7tms |
| MG06738.4 | EAA55081 | 7tms | 7tms | 7tms |
| ^b^MG06755.4 | EAA55098 | 7tms | 7tms |  |
| MG06797.4 | EAA55140 | 7tms | 7tms | 7tms |
| MG07420.4 | EAA53143 | 7tms | 7tms | 7tms |
| ^b^MG07553.4 | EAA53276 | 7tms | 7tms | 7tms |
| ^b^MG07565.4 | EAA53288 | 7tms | 7tms | 7tms |
| MG07806.4 | EAA53529 | 7tms | 7tms |  |
| MG07851.4 | EAA53574 | 7tms |  |  |
| MG07857.4 | EAA53580 | 7tms | 7tms | 7tms |
| ^b^MG07946.4 | EAA53669 | 7tms | 7tms | 7tms |
| MG07987.4 | EAA57018 | 7tms | 7tms | 7tms |
| MG08525.4 | EAA49610 | 7tms | 7tms | 7tms |
| MG08653.4 | EAA51131 | 7tms | 7tms |  |
| MG08803.4 | EAA51281 | 7tms | 7tms | 7tms |
| ^b^MG09022.4 | EAA47892 | 7tms | 7tms |  |
| MG09070.4 | EAA47940 | 7tms | 7tms |  |
| MG09091.4 | EAA47961 | 7tms | 7tms | 7tms |
| MG09416.4 | EAA51399 | 7tms | 7tms | 7tms |
| MG09437.4 | EAA51420 | 7tms | 7tms |  |
| ^b^MG09455.4 | EAA51438 | 7tms | 7tms | 7tms |
| MG09667.4 | EAA48130 |  | 7tms | 7tms |
| ^b^MG09863.4 | EAA53900 | 7tms | 7tms |  |
| MG09865.4 | EAA53902 | 7tms | 7tms | 7tms |
| ^b^MG10050.4 | EAA49886 | 7tms |  |  |
| MG10257.4 | EAA48194 | 7tms | 7tms | 7tms |
| MG10407.4 | EAA51491 |  | 7tms | 7tms |
| MG10430.4 | EAA51514 |  |  | 7tms |
| ^b^MG10438.4 | EAA46744 | 7tms | 7tms |  |
| MG10442.4 | EAA46748 | 7tms | 7tms |  |
| ^b^MG10473.4 | EAA46779 | 7tms | 7tms | 7tms |
| MG10544.4 | EAA46851 | 7tms | 7tms | 7tms |
| MG10571.4 | EAA48312 | 7tms | 7tms | 7tms |
| MG10581.4 | EAA48322 | 7tms | 7tms | 7tms |
| MG10706.4 | EAA46895 | 7tms | 7tms |  |
| MG10747.4 | EAA46936 | 7tms | 7tms |  |
| MG11006.4 | EAA55509 | 7tms | 7tms | 7tms |
|  |  |  |  |  |

^a^See Materials and Method for details regarding the 7tms predictions. In the last three columns “7tms” indicates that the particular program predicted 7tms topology.

^b^N-terminus contains the hydrophobic extracellular CFEM domain which in some cases was counted as a tms in the predictions.
