# Supplementary material for: Novel G-protein-coupled receptor-like proteins in the plant pathogenic fungus Magnaporthe grisea
Source: Genome Biol. 2005 Mar 2;6(3):R24. doi: 10.1186/gb-2005-6-3-r24 (PMC1088943; doi:10.1186/gb-2005-6-3-r24)
Supplement: Additional File 2 — M. grisea-GPCR-like protein classes and N. crassa homologs. [file gb-2005-6-3-r24-S2.doc]

# Supplemental information

# Table S2. *M. grisea*-GPCR-like protein classes and N. *crassa* homologs^a^.

| Class of *M*. *grisea* predicted GPCR | *M*. *grisea* proteins in this class | *N*. *crassa* proteins, matched as best hit with MG protein (BLASTP) | NCU protein E value | NCU Ortholog |
| --- | --- | --- | --- | --- |
|  |  |  |  |  |
| Pheromone receptor STE2 like | MG04711.4 | NCU05758.4 | 1e-69 | Yes |
| Pheromone receptor STE3 like | MG06452.4 | NCU00138.4 | 2e-58 | Yes |
| Class E cAMP receptor like subclass 1 | MG06738.4 | NCU04626.1  (CAD37067; related to GPCR) | 1e-112 | Yes |
| Class E cAMP receptor like subclass 2 |  | NCU09427.1 | 1e-93 |  |
| Class E cAMP receptor like subclass 3 |  | NCU00786.1 | 1e-29 |  |
| MG06738.1 related subclass 1 | MG06797.4  MG06257.4 |  |  |  |
| MG06738.1 related subclass 2 | MG10544.4 |  |  |  |
| MG06738.1 related subclass 3 | MG00326.4 |  |  |  |
| MG06738.1 related subclass 4 | MG00258.4 |  |  |  |
| GPCR GPR1 | MG08803.4 | NCU06312.1 | 4e-64 | Yes |
| GPCR STM1 | MG04698.4  MG02855.4 | NCU00300.1  NCU09195.1  (CAD79648; conserved hypo) | 1e-109  1e-103 | Yes  Yes |
| GPCR MPR like subclass 1 | MG05072.4 | NCU04987.1 | 1e-111 | Yes |
| GPCR MPR subclass 2 | MG09091.4 |  |  |  |
| GPCR MPR subclass 2 | MG04679.4 | NCU03238.1 | 1e-171 | Yes |
| PTH11 receptor related subclass 1 | MG05871.4 (PTH11) |  |  |  |
| PTH11 receptor related subclass 2 | MG10473.4  MG07553.4  MG06755.4  MG09022.4  MG07565.4  MG07946.4 | NCU06531.1 | 3e-70 | Yes |
| PTH11 receptor related subclass 3 | MG11006.4  MG09070.4  MG07806.4  MG03584.4 | NCU00700.1 |  | Yes |
| PTH11 receptor related subclass 4 | MG05214.4 |  |  |  |
| PTH11 receptor related subclass 5 | MG09863.4 |  |  |  |
| PTH11 receptor related subclass 6 | MG10407.4 |  |  |  |
| PTH11 receptor related subclass 7 | MG10571.4 |  |  |  |
| PTH11 receptor related subclass 8 | MG01867.4 |  |  |  |
| PTH11 receptor related subclass 9 | MG09455.4  MG10050.4 |  |  |  |
| PTH11 receptor related subclass 10 | MG05352.4 |  |  |  |
| PTH11 receptor related subclass 11 | MG07420.4 |  |  |  |
| PTH11 receptor related subclass 12 | MG10442.4 | NCU08624.1 | 6e-48 | Yes |
| PTH11 receptor related subclass 13 | MG02160.4 |  |  |  |
| PTH11 receptor related subclass 14 | MG02001.4 |  |  |  |
| PTH11 receptor related subclass 15 | MG10257.4  MG01905.4  MG07987.4 | NCU05854.1 | 1e-66 | Yes |
| PTH11 receptor related subclass 16 | MG10438.4 |  |  |  |
| PTH11 receptor related subclass 17 | MG06171.4 |  |  |  |
| PTH11 receptor related subclass 18 | MG07851.4 |  |  |  |
| PTH11 receptor related subclass 19 | MG04935.4 | NCU07649.1 | 1E-41 | Yes |
| PTH11 receptor related subclass 20 | MG05386.4 |  |  |  |
| PTH11 receptor related subclass 21 | MG09865.4 | NCU07591.1 | 4E-69 | Yes |
| PTH11 receptor related subclass 22 | MG05514.4 |  |  |  |
| PTH11 receptor related subclass 23 | MG06535.4 | NCU09201.1 |  |  |
| PTH11 receptor related subclass 24 | MG01190.4 |  |  |  |
| PTH11 receptor related subclass 25 | MG10581.4 |  |  |  |
| PTH11 receptor related subclass 26 | MG03009.4 |  |  |  |
| PTH11 receptor related subclass 27 | MG10747.4 | NCU07538.1 |  |  |
| PTH11 receptor related subclass 28 | MG03935.4  MG04682.4 |  |  |  |
| PTH11 receptor related subclass 29 | MG09416.4 |  |  |  |
| PTH11 receptor related subclass 30 | MG02692.4 |  |  |  |
| PTH11 receptor related subclass 31 | MG07857.4 |  |  |  |
| PTH11 receptor related subclass 32 | MG00826.4 |  |  |  |
| PTH11 receptor related subclass 33 | MG06624.4 |  |  |  |
| PTH11 receptor related subclass 34 | MG00435.4 |  |  |  |
| PTH11 receptor related subclass 35 | MG08653.4 |  |  |  |
| PTH11 receptor related subclass 36 | MG10706.4 |  |  |  |
| PTH11 receptor related subclass 37 | MG04170.4 |  |  |  |
| PTH11 receptor related subclass 38 | MG08525.4 |  |  |  |
| PTH11 receptor related subclass 39 | MG00277.4  MG02365.4 | NCU05189.1 | 2E-42 | Yes |
| PTH11 receptor related subclass 40 | MG06595.4 |  |  |  |
| PTH11 receptor related subclass 41 | MG06084.4 | NCU05307.1 | 1E-77 | Yes |
| PTH11 receptor related subclass 42 | MG09437.4 |  |  |  |
| PTH11 receptor related subclass 43 | MG01890.4 | NCU08447.1 |  | Yes |
| PTH11 receptor related subclass 44 | MG01871.4 |  |  |  |
| PTH11 receptor related subclass 45 | MG03794.4 |  |  |  |
| PTH11 receptor related subclass 46 | MG01884.4 |  |  |  |
| PTH11 receptor related subclass 47 | MG09667.4 | NCU02903.1 |  |  |
| PTH11 receptor related subclass 48 |  | NCU09823.1 |  |  |
| PTH11 receptor related subclass 49 |  | NCU09796.1 |  |  |
| PTH11 receptor related subclass 50 |  | NCU04106.1 |  |  |
| PTH11 receptor related subclass 51 |  | CAC28570? |  |  |
| PTH11 receptor related subclass 52 |  | NCU04931.1 |  |  |
| PTH11 receptor related subclass 53 |  | NCU08718.1 |  |  |
| PTH11 receptor related subclass 54 | MG09061.4 | NCU09022.1 |  |  |
| PTH11 receptor related subclass 55 |  | NCU05101.1 |  |  |
| PTH11 receptor related subclass 56 |  | NCU08431.1  NCU08429.1 |  |  |
| PTH11 receptor related subclass 57 |  | NCU06891.1 |  |  |
| PTH11 receptor related subclass 58 |  | NCU05187.1 |  |  |
| PTH11 receptor related subclass 59 |  | NCU05829.1 |  |  |
| MG00532.1 like | MG00532.4 | NCU03253.1 | 1e-114 | Yes |
|  |  |  |  |  |

^a^The *M*. *grisea* and *N*. *crassa* GPCR-like proteins were clustered according to the criteria used to cluster *M*. *grisea* paralogs (Table 1). The *N*. *crassa* proteins with the best hit in bi-directional BLAST searches were defined as orthologs.

GenBank accession numbers are also included for the *N*. *crassa* proteins that have an entry in GenBank (nr) database.
