# Supplementary material for: Novel G-protein-coupled receptor-like proteins in the plant pathogenic fungus Magnaporthe grisea
Source: Genome Biol. 2005 Mar 2;6(3):R24. doi: 10.1186/gb-2005-6-3-r24 (PMC1088943; doi:10.1186/gb-2005-6-3-r24)
Supplement: Additional File 3 — M. grisea CFEM-containing proteins that may be membrane associated or secreted. [file gb-2005-6-3-r24-S3.doc]

# Supplemental information

# Table S3. *M*. *grisea* CFEM containing proteins that may be membrane associated or secreted.

|  | CFEM containing *M*. *grisea* protein | Best hit (BLASTP)Protein E value | |
| --- | --- | --- | --- |
|  |  |  |  |
| 1 | MG01149.4 | *C*. *albicans* RBT5 | 6e-07 |
| 2 | MG01872.4 | *N*. *crassa* conserved | 2e-05 |
| 3 | MG05456.4 | *N*. *crassa* conserved | 0.2 |
| 4 | MG05531.4 | *M*. *grisea* ACI1 | 0 |
| 5 | MG07005.4 | *N*. *crassa* related to chitinase | 2e-15 |
| 6 | MG08321.4 | *C*. *immitis* proline rich antigen | 0.048 |
| 7 | MG09570.4 | - | - |
|  |  |  |  |
